# Case Report: Minigene assays reveal a novel *DNAAF6* intronic variant as the key etiology for primary ciliary dyskinesia

**DOI:** 10.3389/fgene.2025.1692658

**Published:** 2025-12-18

**Authors:** Yupeng Long, Huixian Li, Haoyue Yu, Qian Yi, Xiong Meng, Xi Wang, Qinqin Ren, Dongyan Ding, Haidong Li, Fenglan Zhang, Hao Qiu, Xuemei Yang

**Affiliations:** 1 Laboratory and Blood Transfusion Department of Jiangbei Campus, The First Affiliated Hospital of Army Medical University (The 958th hospital of Chinese People’s Liberation Army), Chongqing, China; 2 Center for Clinical Genetics and Genomics, Dian Diagnostics Group Co., Ltd., Hangzhou, Zhejiang, China; 3 Respiratory and Critical Care Medicine Department of Jiangbei Campus, The First Affiliated Hospital of Army Medical University (The 958th hospital of Chinese People’s Liberation Army), Chongqing, China

**Keywords:** case report, DNAAF6 gene, male infertility, minigene, primary ciliary dyskinesia

## Abstract

**Background:**

Primary ciliary dyskinesia (PCD), a rare hereditary disorder characterized by impaired ciliary motility, is frequently linked to infertility. Elucidating PCD’s genetic basis is critical for accurate diagnosis and clinical management. However, research on the pathogenicity of intronic variants in non-classical splice regions of *DNAAF6*—a newly identified PCD-associated gene in recent years—remains scarce.

**Case presentation:**

A proband with primary ciliary dyskinesia (PCD) and male infertility underwent whole exome sequencing (WES), revealing a novel hemizygous intronic variant in the *DNAAF6* gene (NM_173494.2: c.515 + 3_515+6del, Clinvar Variation ID:4075425). Sanger sequencing confirmed this variant in his affected brother. A minigene splicing assay showed that the variant caused exon six skipping, leading to a frameshift. Transmission electron microscopy (TEM) analysis of sperm flagella indicated absent outer and inner dynein arms, resulting in flagellar immotility.

**Conclusion:**

This study identifies and confirms a novel pathogenic intronic variant of *DNAAF6* in a Chinese PCD family, expanding the *DNAAF6* mutational spectrum. It also emphasizes the critical role of minigene-based functional validation in interpreting variants of uncertain significance (VUS).

## 1 Introduction

Primary ciliary dyskinesia (PCD) represents a rare, inherited disorder specifically affecting motile cilia. This condition is precipitated by genetic variations that give rise to abnormalities in both the structure and function of cilia. Consequentially, these aberrations lead to disorders in the movement of ciliary epithelium within multiple organs. As a result, organs harboring ciliary tissue experience dysfunction, thereby posing a significant threat to human health. The reported incidence of PCD ranges approximately from 0.0025% to 0.0050% (Goutaki et al., 2016). Affected individuals typically manifest signs of PCD either at birth or during the initial few months of life. Nevertheless, due to substantial diagnostic complexities, a subset of PCD cases remains undiagnosed until adulthood. Manifestations such as chronic airway infections, bronchiectasis secondary to chronic airway infections, and chronic sinusitis are commonly discernible in early childhood (Zariwala et al., 1993). Moreover, infertility, chronic or recurrent ear infections, and situs inversus are among the characteristic symptoms associated with PCD (Zariwala et al., 1993).

PCD is genetically heterogeneous as there are thought to be over 200 genes involved in ciliogenesis (Damseh et al., 2017). These genes encode proteins involved in axonemal motors, structure, and regulation, or ciliary assembly and preassembly. To date, approximately 54disease-causing genes accounting for approximately 70% of PCD cases have been implicated in humans (Zariwala et al., 1993; Despotes et al., 2024). PCD is typically inherited in an autosomal recessive manner except for *FOXJ1* (MIM: 602291) (which is autosomal dominant) and *OFD1* (MIM: 311200) and *RPGR* (MIM: 312610) (which are X-linked) (Zariwala et al., 1993; Ferrante et al., 2006; Moore et al., 2006). Here, we describe another instance of X-linked PCD, which is attributed to mutations in the *DNAAF6* (MIM: 300933).

*DNAAF6* represents a recently identified X-linked gene implicated in causing PCD. It is a protein-coding gene that encodes a highly conserved protein. This protein is uniquely present in species that possess motile cilia or flagella with dynein arms and in those organisms that rely on intraflagellar transport (IFT) for ciliary assembly (Olcese et al., 2017). In 2014, it was initially discovered that *DNAAF6* plays a crucial role in the cytoplasmic preassembly of axonemal dynein within mouse sperm (Dong et al., 2014). By 2017, two independent research groups had identified that pathogenic variants in *DNAAF6* are associated with X-linked primary ciliary dyskinesia in the human population (Olcese et al., 2017; Paff et al., 2017). According to the Human Protein Atlas, human *DNAAF6* is reported to be expressed in ciliated tissues. Among these, the highest levels of expression are observed in the fallopian tube, testis, and respiratory tract. To date, dozens of variants in *DNAAF6* that result in PCD have been documented, including genomic deletion variants (GenBank: NM_001169154.1, 0.044-Mb, 0.185-Mb, 1.93-Mb, 3.27-Mb and 3.73-Mb deletions) (Olcese et al., 2017; Huang et al., 2021; Xu et al., 2022); frameshift variants (c.357_363del [p.Val120LeufsTer6], c.263_268delinsG [p.Ile88ArgfsTer12], c.489_492del [p.Ile164LeufsTer11], c.319_329del [p.Glu108ValfsTer2]) (Olcese et al., 2017; Paff et al., 2017); nonsense variants (c.355C>T [p.Gln119Ter], c.127G>T [p.Glu43Ter], c.266G>A [p.Trp89Ter], c.511C>T [p.Gln171Ter]) (Olcese et al., 2017; Paff et al., 2017); missense variants (c.397G>T [p.Asp133Tyr], c.290G>T [p.Gly97Val]) (Olcese et al., 2017; Wang et al., 2020). Notably, to the best of current knowledge, no intronic variation within *DNAAF6* has been reported.

In the present study, a hitherto unreported intronic variant was uncovered. Investigation demonstrated that this variant gives rise to PCD by exerting an impact on the assembly of axonemal dynein arms.

## 2 Materials and methods

### 2.1 Subjects

The patient was a 35-year-old male who was hospitalized due to aggravated cough and back pain. He had a medical history of bronchiectasis and chronic sinusitis. Additionally, he had a 20-year smoking history. He fathered a daughter via assisted reproductive technology due to immotile sperm.

The patient’s parents were in good health. However, his brother presented similar respiratory manifestations, including dextrocardia, chronic sinusitis, and chronic productive cough. The patient’s uncle with similar respiratory manifestations passed away.

### 2.2 Whole exome sequencing (WES) and data analysis

Genomic DNA (gDNA) was extracted from the patient’s peripheral blood with the QIAamp DNA Blood Midi Kit (Qiagen, Germany) following standard protocols. Whole exome sequencing was performed for the patient by DIAN Diagnostics Corporation (Hangzhou, China) using the IDT xGen Exome Research Panel v1.0 (Integrated DNA Technologies, USA) with 150-bp paired-end reads, on an Illumina NovaSeq 6,000 system (Illumina, USA); the sequencing achieved 99.46% coverage at a depth of >20×. Sequences were aligned against the human reference genome hg19, with variants named via the Genome Analysis Toolkit (GATK) and annotated using SnpEff and VEP. Identified variants were verified by Sanger sequencing, and data interpretation complied with the American College of Medical Genetics and Genomics (ACMG) guidelines, which classify variants into five categories: benign, likely benign, variant of uncertain significance (VUS), likely pathogenic, and pathogenic (Richards et al., 2015).

### 2.3 Sanger sequencing

gDNA of the patient and relatives were amplified and sequenced with specific primer pairs, as follows: Primer1 Forward (*DNAAF6* c.515 + 3_515+6del) 5′-AAG​GTC​CCC​AAC​TCT​AAC​AGG-3′ and Primer1 reverse (*DNAAF6* c.515 + 3_515+6del) 5′-ACT​GCA​TCT​GGG​TGT​CAA​GAA​T-3’. An ABI 3500Dx automated sequencer (Applied Biosystems, USA) was used to analyze the PCR products.

### 2.4 Minigene splicing experiment

To assess the impact of the c.515 + 3_515+6del variant on splicing, wild-type and mutant minigenes were constructed, encompassing *DNAAF6* (NM_173494) exon 5, exon 6, exon 7, with their flanking intronic sequences. Target segments were amplified by high-fidelity PCR (TOYOBO BIOTECH, Japan) from genomic DNA of a hemizygous patient and a control using the following primers:

Exon 5: F: 5′-AAG​CTT​GGT​ACC​GAG​CTC​GGA​TCC​GTA​TGA​GAT​TAT​ATT​CAG​ACA​GCA​GGT​G-3’; R: 5′-AGG​TGT​TAT​GGA​GTC​ATT​ACG​AGA​AAT​ACA​GAA​ATA​AG-3’

Exon 6 and flanking introns: F: 5′-GTA​ATG​ACT​CCA​TAA​CAC​CTG​AAC​TCC​CAC​ACC​TCC-3’; R: 5′-GTA​GTT​TTT​GGA​GAT​GAT​TGT​TCT​GTG​AGG​ATA​CAT​T-3’

Exon 7: F: 5′-CAA​TCA​TCT​CCA​AAA​ACT​ACA​CCT​GTG​GTA​GAG​ACT​C-3’; R: 5′-TTA​AAC​GGG​CCC​TCT​AGA​CTC​GAG​TCA​GAA​GAA​ATT​AGC​AAT​ATC​TAA​CTC​TC-3’

The PCR fragments were cloned into the pMini-CopGFP vector (BamHI/XhoI) using a seamless cloning kit (Vazyme Biotech, China). HEK293T cells were maintained in DMEM-F12/10% FBS and transfected with 4 µg of each minigene using Lipofectamine® 2000 (Thermo Fisher Scientific, USA). Total RNA was extracted 48 h post-transfection. cDNA was synthesized with random primers (HiScript II kit, Vazyme Biotech, China), followed by PCR amplification using vector-specific primers (MiniRT-F: 5′-GGC​TAA​CTA​GAG​AAC​CCA​CTG​CTT​A-3’; DNAAF6-RT-R: 5′-TCA​GAA​GAA​ATT​AGC​AAT​ATC​TAA​CTC​TC-3′) and Kod-Plus-Neo polymerase (TOYOBO BIOTECH, Japan). Splicing patterns were analyzed by 5% polyacrylamide gel electrophoresis and sequencing of the amplified cDNA fragments.

### 2.5 Protein structural analysis

SWISS-MODEL software was used to analyze the structure of the mutant DNAAF6 protein (NP_775765.1) and PyMol software was used to visualize the structure.

## 3 Case presentation

### 3.1 Clinical characteristics of the patient

The patient is a 35-year-old male who has experienced frequent cough, sputum production, and nasal congestion since childhood, occasionally accompanied by fever. Symptoms can be alleviated with antipyretics and cephalosporins. He had a medical history of bronchiectasis and chronic sinusitis. After marriage, he experienced fertility difficulties, and hospital tests showed immotile sperm. He fathered a daughter through artificial insemination. Over the past 6 months, the patient’s cough and sputum production have worsened, accompanied by back pain, leading him to seek medical attention. After hospitalization, the patient received aggressive anti-inflammatory treatment, resulting in improvements in both his cough and lung function.

**FIGURE 1:**
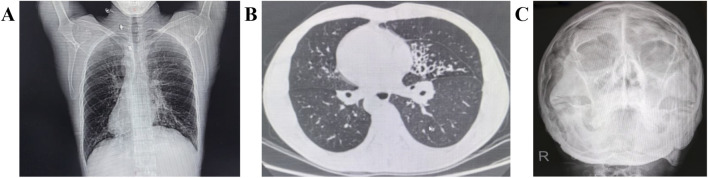
Clinical Manifestations of the Proband with PCD. Computed tomography scans show dextrocardia **(A)** and bronchiectasis **(B)**. X-ray of the paranasal sinus shows chronic inflammation of bilateral maxillary sinuses **(C)**.

His symptoms are consistent with the typical manifestations of primary ciliary dyskinesia (PCD). X-ray examination of the paranasal sinuses revealed chronic inflammation in both maxillary sinuses, and CT scans confirmed dextrocardia and bronchiectasis (Figure 1). Color Doppler ultrasonography identified total situs inversus, with the liver and gallbladder on the left and the spleen on the right. A diagnostic work-up according to European Respiratory Society (ERS) guidelines confirmed the PCD diagnosis (Leigh et al., 2013). Routine semen analysis showed mixed results: concentration and total sperm number were normal, while most of the indicated reduced sperms were immotility (Supplementary Table S1). Supplementary Material include videos demonstrating complete immotility of the patient’s sperm flagella (Supplementary Data 2) compared to a control subject (Supplementary data 1).

The patient’s family history revealed that his parents were healthy, but his brother exhibited similar respiratory symptoms, including dextrocardia and chronic sinusitis. An uncle with comparable symptoms had passed away.

### 3.2 Identification of *DNAAF6* variant in the family

Whole exome sequencing (WES) and bioinformatic analyses were performed to determine the genetic etiology in the patient suspected of having ciliary dyskinesia. Single nucleotide variants (SNVs) and insertions/deletions (InDels) were called, then filtered and annotated via NCBI dbSNP (https://www.ncbi.nlm.nih.gov/snp/), 1000 Genomes Project, gnomAD (https://gnomad.broadinstitute.org/), and the in-house DIAN Diagnostics database. Missense variant impact was predicted using dbNSFP (https://www.dbnsfp.org/) (SIFT, Polyphen2, LRT, MutationTaster, PhyloP, etc.) as well as REVEL and ClinPred. SpliceAI and dbscSNV were used for splice variant analysis. Variant pathogenicity was annotated based on ClinVar (https://www.ncbi.nlm.nih.gov/clinvar), the Human Gene Mutation Database (HGMD), and the ACMG Standards and Guidelines for the Interpretation of Sequence Variants (Richards et al., 2015). Subsequently, with primary ciliary dyskinesia (PCD) as the phenotype for variant filtering, only one hemizygous intronic variant, *DNAAF6*:c.515 + 3_515+6del (ClinVar Variation ID: 4075425), was identified among PCD-related genes, which was initially classified as uncertain significance (PM2).

**FIGURE 2:**
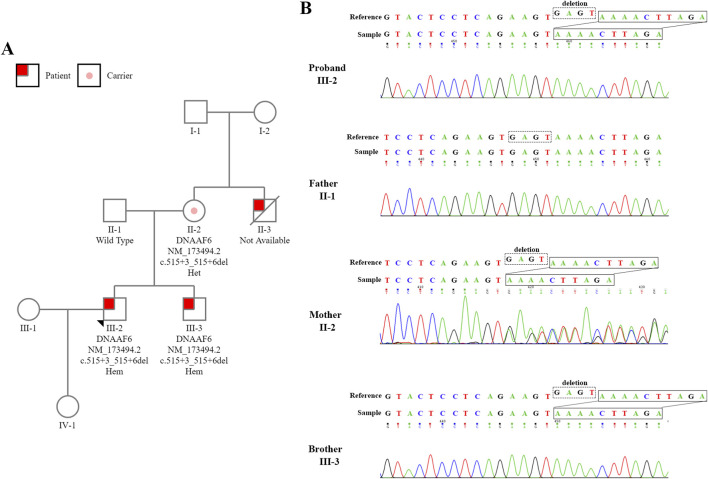
Pedigree Analysis and Sanger Sequencing Validation of Patients Harboring *DNAAF6* Variants. **(A)** A hemizygous intronic variant within the *DNAAF6* gene, which is located on the X chromosome, was detected in the family. PCD-affected siblings are represented by shaded red squares, and the unaffected siblings are denoted by shaded white squares or circles. Female carriers with the heterozygous variant are indicated by pink circles. **(B)** Sanger sequencing successfully identified the *DNAAF6* variants in the proband, his parents, and his brother.

Sanger sequencing was subsequently employed to validate the identified variant in the patient (Ⅲ-2), as well as in his parents and his brother (Ⅲ-3) (Figure 2). His mother (Ⅱ-2) was determined to be a heterozygous carrier, while his affected brother also carried the hemizygous variant. His father (Ⅱ-1) with a normal phenotype was wild type. A pedigree analysis was conducted to explore the potential of an X-linked recessive inheritance pattern within this patient’s family (Figure 2). The proband’s uncle, who had no children and had a history of similar respiratory symptoms and infertility, unfortunately, was not available for blood sampling for the pedigree study due to his death at the age of 40. The overall results strongly suggested that the variant in *DNAAF6* was associated with both PCD and male infertility.

### 3.3 Minigene splicing assay

**FIGURE 3:**
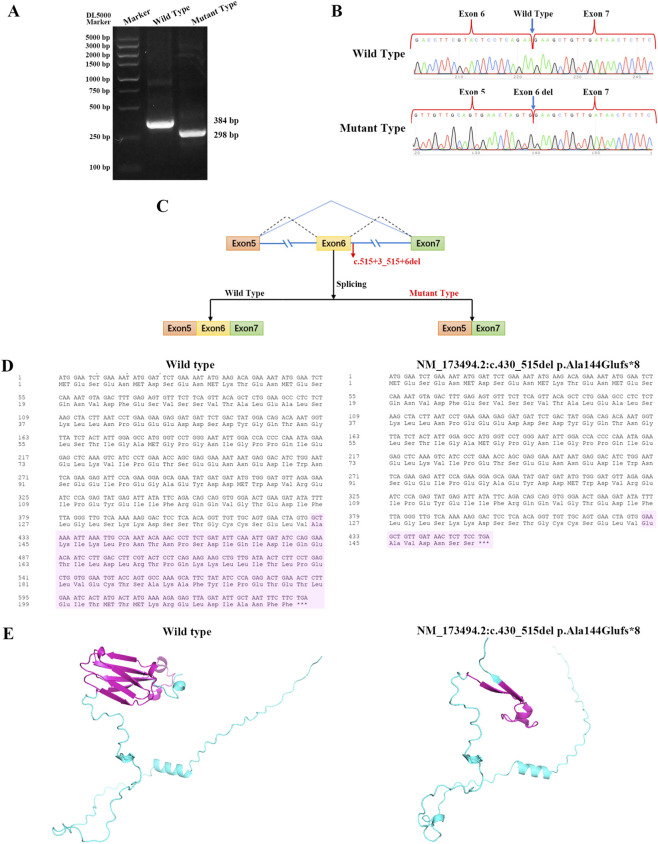
Representation of the minigene splicing assays for the *DNAAF6*:c.515 + 3_515+6del variation. **(A)** The outcome of 1.5% agarose gel electrophoresis of the reverse transcription-polymerase chain reaction (RT-PCR) products derived from the wild-type and variant minigene constructs (*DNAAF6*:c.515 + 3_515+6del) is depicted. **(B)** Exon six skipping was detected through direct sequencing of the amplification products obtained from the minigene constructs. **(C)** A schematic illustration of the splicing events of the *DNAAF6* minigene is provided. **(D)** Prediction of the shortening of both the messenger RNA (mRNA) and amino acid sequences in the mutant type, consequent to exon six skipping, is shown. **(E)** Prediction of the protein’s three-dimensional structure demonstrated a shortening of the amino acid sequence, along with a reduction in β-folding and α-helix elements.

To elucidate the impact of the c.515 + 3_515+6del variant on splicing, both wild-type and mutated minigenes were meticulously constructed. The amplification of the *DNAAF6* cDNA from the wild-type construct yielded a single fragment with the anticipated size of 384 base pairs (bp) (Figure 3A). In comparison to the wild-type, a relatively smaller product of approximately 300 bp was detected in the mutant-type sample (Figure 3A). Sanger sequencing of the cDNA-amplified products demonstrated that the *DNAAF6*:c.515 + 3_515+6del variant results in the deletion of 86 bp and skipping of exon 6 (Figures 3B,C). The mutant mRNA and amino acid sequences following exon six skipping were predicted (Figure 3D). The variant exerts a complete modifying effect on the splicing of exon 6, leading to a frameshift mutation (p.Ala144GlufsTer8) and the introduction of a premature stop-codon. Prediction of the protein’s three-dimensional structure indicated a shortening of the amino acid sequence, along with a reduction in β-folding and α-helix content. These changes may potentially lead to alterations in the protein’s conformation and stability (Figure 3E).

### 3.4 Genetic variation analysis of *DNAAF6* gene in the proband

According to the ACMG standards and guidelines for the interpretation of sequence variants (Richards et al., 2015), the initial classification for the c.515 + 3_515+6del variant was uncertain significance (PM2). Notably, results from the minigene splicing experiment provided crucial insights. *In vitro* functional studies demonstrated a damaging effect on the gene, thus fulfilling the PS3 criterion. Considering the cumulative evidence of PM2 and the *in vitro* functional data (PS3), it is proposed that the classification of the c.515 + 3_515+6del variant within the *DNAAF6* gene be upgraded to “likely pathogenic”.

Based on the comprehensive analysis of the patient’s clinical manifestations and the results of genetic testing, it is highly hypothesized that the *DNAAF6* variant is the etiological factor underlying PCD in the proband and his family members.

### 3.5 Structural analyses validate the dynein arm defect in sperm flagella

**FIGURE 4:**
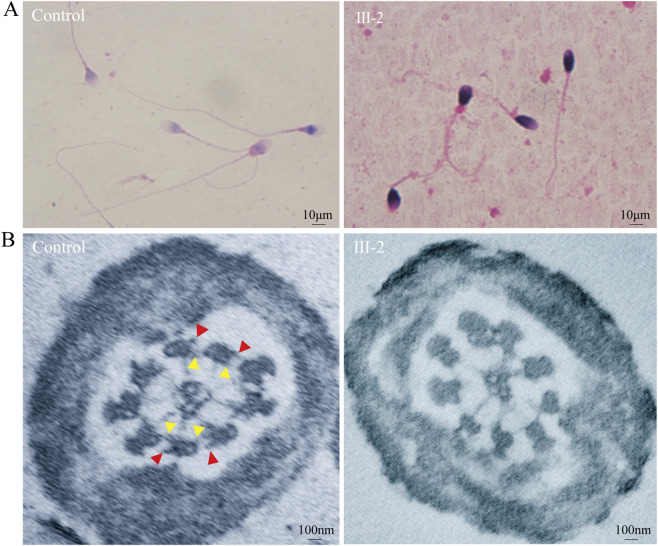
Abnormal morphology and ultrastructure of mutant spermatozoa. **(A)** H&E staining of spermatozoa from both patient (III-2) and control indicated short tails and coiled tails of *DNAAF6* mutant spermatozoa. Scale bars, 10 μm. **(B)** Ultrastructural analysis of sperm flagella in patient (III-2) and control. Cross-sectional views of the principal piece of sperm flagella were obtained from both control subjects and the patient. TEM analysis of cilia cross-sections from a healthy control revealed the characteristic arrangement. The ODA, denoted by red arrowheads, and IDA, marked by yellow arrowheads, were clearly distinguishable. In contrast, while the overall axonemal structures were retained in the spermatozoa of the patient with the *DNAAF6* variant, a specific loss of both ODAs and IDAs was evident. The ultrathin sections were stained using uranyl acetate and lead citrate. Scale bars, 100 nm.

H&E staining of the mutated spermatozoa indicated that the majority of sperm exhibited abnormal tail morphologies, including short tails and coiled tails (Figure 4A). To evaluate the ultrastructure of sperm flagella, additional TEM analyses were conducted (Figure 4B). TEM analysis of samples from a healthy control clearly presented the cross-sectional arrangement of the flagellum. It also provided an image of the key components of the 9 + 2 motile axoneme, such as the outer dynein arms (ODA) and inner dynein arms (IDA). Conversely, in the cross-sections of the proband’s sperm flagella, although a normal 9 + 2 architecture was observable, there was a distinct absence of both the outer and inner dynein arms.

## 4 Discussion

In this study, we identified a novel intronic variant (c.515 + 3_515+6del) in the *DNAAF6* gene in a Chinese family with PCD and male infertility using WES and Sanger sequencing. Notably, this represents the first reported intronic variant of the *DNAAF6* gene. Minigene splicing assays yielded conclusive evidence confirming the pathogenicity of this variant, demonstrating that the c.515 + 3_515+6del variant induces exon six skipping, thereby resulting in a frameshift mutation. TEM analysis of the patient’s sperm further confirmed the absence of both ODA and IDA in the sperm flagella. Collectively, these findings indicate that the identified intronic variant may be responsible for severe PCD and infertility, with the underlying mechanism likely involving disruption of early axonemal dynein arm assembly.

*DNAAF6* (also known as PIH1D3) is a dynein axonemal assembly factor containing a single PIH domain. It forms a co-chaperone complex with DNAAF2 and DNAAF4, and modulates the cytoplasmic preassembly of dynein arms through interactions with DNAI2 and HSP90 (Olcese et al., 2017; Mali et al., 2018). Since the first association of *DNAAF6* with ciliary dyskinesia in humans in 2017, 15 probands from seven publications have been reported, harboring 15 distinct variants associated with outer and inner dynein arm defects. These variants include four nonsense mutations, four frameshift mutations, two missense mutations, and five severe deletions (Olcese et al., 2017; Paff et al., 2017; Wang et al., 2020; Aprea et al., 2021; Huang et al., 2021; Gardner et al., 2024; Thomas et al., 2024). With the exception of the Gly97Val missense mutation, all other identified variants are localized within the PIH domain or are predicted to result in deletions of the PIH domain. Minigene experiments demonstrated that the novel intronic variant (c.515 + 3_515+6del) identified in this study leads to an 86-bp deletion, which is predicted to convert alanine at position 144 of DNAAF6 to glutamic acid, followed by a stop codon eight amino acids later. The affected amino acids are precisely located within the core PIH domain. Three-dimensional protein structure predictions revealed a reduction in β-sheet and α-helix content, suggesting that this variant may potentially cause conformational and stability changes in the PIH domain. These findings indicate that intronic variants constitute a non-negligible category of pathogenic variants in *DNAAF6*, and that variants located in the PIH domain may represent a hotspot mutation region for the *DNAAF6* gene.

Except for the two reported missense variants, the null variants reported to date suggest that the pathogenic mechanism of *DNAAF6* variants may involve X-linked loss-of-function. According to the ACMG classification system (Richards et al., 2015), loss-of-function variations of the *DNAAF6* gene, including nonsense, frameshift, and those in canonical splicing regions, can be unequivocally classified as at least likely pathogenic (class 4) in the context of PCD. In contrast, without functional characterization, variants affecting deeper intronic regions cannot be classified higher than class 3 (variants of uncertain significance [VUS]). Splice prediction tools (https://rddc.tsinghua-gd.org/zh/tool/rna-splicer) predicted that the novel intronic variant (c.515 + 3_515+6del) identified in this study may have two outcomes: either activating a novel splice donor site in intron 6 (c.515 + 487), inserting 487 bp and causing extension of exon 6, or deleting 86 bp and triggering exon six skipping. However, the manner in which this variant affects splicing and the specific splicing outcome remained unclear. Ultimately, minigene splicing assays revealed that the intronic variant causes exon six skipping, which is predicted to induce a frameshift, thereby confirming the second predicted splicing pattern. Considering the process of nonsense-mediated mRNA decay (NMD), this frameshift is expected to result in the production of a truncated protein or a complete absence of protein synthesis. Based on these results, we were able to reclassify this variant as pathogenic according to the ACMG classification. In routine diagnostic practice, computational simulation tools can predict the potential effects of mutations on splicing; however, their results are not highly reliable. Minigene splicing assays remain the most informative and reliable method for assessing the functional impact of intronic VUS. This achievement will assist clinicians in formulating appropriate strategies for patient counseling and management.

The observation of ciliary morphology and function in PCD patients currently serves as the gold standard for the clinical diagnosis of PCD, with TEM as the preferred method (Lucas et al., 2017). Typically, nasal brushing or bronchoscopic biopsy is used to obtain tissue samples, but these procedures are invasive and cause discomfort to patients. In the present study, we investigated the flagella of the patient’s sperm cells. Our results demonstrated that the ultrastructural defects in spermatozoa with *DNAAF6* mutations are indistinguishable from those reported in cilia with *DNAAF6* mutations (Olcese et al., 2017; Paff et al., 2017; Wang et al., 2020; Huang et al., 2021; Xu et al., 2022). This finding suggests that for male PCD patients, clinical practice may prioritize utilizing ultrastructural examination of sperm flagella combined with pathogenic gene analysis for PCD diagnosis. Our findings also broaden the spectrum of *DNAAF6* variants associated with PCD, particularly in the Chinese population. In comparison to Western populations, relevant genetic data in the Chinese population remain relatively limited. Given that both respiratory cilia and sperm flagella rely on a conserved dynein machinery, the association between this variant and infertility further underscores the pleiotropic effects of ciliary dysfunction. Future studies with larger cohorts are needed to determine the prevalence of this gene in Asian populations and its penetrance across different clinical manifestations of PCD.

A limitation of the present study is the inability to conduct comprehensive clinical and imaging evaluations of the proband’s family members due to their refusal to participate, which precludes a more holistic understanding of the phenotypic spectrum. Furthermore, while minigene assays effectively recapitulate splicing effects, *in vivo* studies, such as those utilizing model organisms, would likely provide deeper insights into how this variant impacts ciliary structure and function, as well as the precise mechanisms by which the truncated DNAAF6 protein disrupts dynein arm assembly.

In conclusion, this study not only identifies a pathogenic *DNAAF6* variant in a Chinese family with PCD but also underscores the critical role of functional validation in interpreting VUS. As genomic sequencing becomes increasingly integrated into routine clinical practice, such functional validation strategies will be pivotal for translating genetic findings into precise diagnoses and, ultimately, for enhancing the management of PCD and related ciliopathies.

## Data Availability

The datasets presented in this article are not readily available because of ethical and privacy restrictions. Requests to access the data that support the findings of this study should be directed to the corresponding author.
