# Supplementary material for: Case Report: Minigene assays reveal a novel DNAAF6 intronic variant as the key etiology for primary ciliary dyskinesia
Source: Front Genet. 2025 Dec 18;16:1692658. doi: 10.3389/fgene.2025.1692658 (PMC12755853; doi:10.3389/fgene.2025.1692658)
Supplement: Supplementary file 2 [file Table1.docx]

Supplementary Material

Case Report: Minigene assays reveal a novel *DNAAF6* intronic variant as the key etiology for primary ciliary dyskinesia

Yupeng Long^1^, Huixian Li^2^, Haoyue Yu^3^, Qian Yi^3^, Xiong Meng^3^, Xi Wang^3^, Qinqin Ren^3^, Dongyan Ding^3^, Haidong Li^3^, Fenglan Zhang^2^, Hao Qiu^2^, Xuemei Yang^3*^

*** Correspondence:** Xuemei Yang.renew_y@hotmail.com

# Supplementary Tables

Table S1. Semen parameters of the proband with *DNAAF6* variant

| Patient | Volume (ml) | Concentration (10^6^/ml) | Total sperm number (10^6^) | pH | PR (%) | NP (%) | PR + NP (%) | Normal sperm morphology (%) |
| --- | --- | --- | --- | --- | --- | --- | --- | --- |
| Patient | 2.0 | 17.2 | 34.4 | 7.0 | 0.0 | 0.4 | 0.4 | 0.1 |
| Lower reference limits | 1.5  (1.4–1.7) | 15  (12–16) | 39  (33–46) | ≥7.2 | 32  (31–34) | – | 40  (38–42) | 4 |

PR, progressive motility, NP, non-progressive motility
